# Effectiveness and safety of ventriculoperitoneal shunt versus lumboperitoneal shunt for communicating hydrocephalus: A systematic review and meta‐analysis with trial sequential analysis

**DOI:** 10.1111/cns.14086

**Published:** 2023-01-17

**Authors:** Yi‐Jen Ho, Wen‐Chun Chiang, Hsin‐Yi Huang, Shinn‐Zong Lin, Sheng‐Tzung Tsai

**Affiliations:** ^1^ Department of Neurosurgery, Hualien Tzu Chi Hospital Buddhist Tzu Chi Medical Foundation Hualien Taiwan; ^2^ Department of Chest Medicine, Hualien Tzu Chi Hospital Buddhist Tzu Chi Medical Foundation Hualien Taiwan; ^3^ Department of Medical Research, Hualien Tzu Chi Hospital Buddhist Tzu Chi Medical Foundation Hualien Taiwan; ^4^ School of Medicine Tzu Chi University Hualien Taiwan

**Keywords:** communicating hydrocephalus, lumboperitoneal shunt, meta‐analysis, ventriculoperitoneal shunt

## Abstract

**Introduction:**

The current standard surgical treatment for cerebrospinal fluid diversion is a ventriculoperitoneal shunt (VPS) implantation. Lumboperitoneal shunts (LPS) are an alternative treatment for communicating hydrocephalus. Prior studies comparing these two included a limited number of participants.

**Methods:**

We performed a meta‐analysis determined the treatment failure, complications and effectiveness of lumboperitoneal shunt for communicating hydrocephalus. We reviewed studies with clinical and imaging diagnoses of communicating hydrocephalus, all causes and subtypes of communicating hydrocephalus, and studies that analyzed the primary and secondary outcomes listed below. We included randomized controlled trials (RCTs), non‐RCTs and retrospective studies. We performed the meta‐analysis in R, using a random‐effects model and reporting 95% confidence intervals.

**Results:**

Data from 25 studies, including 3654 patients, were analyzed. The total complication rates were 12.98% (188/1448) for lumboperitoneal shunt and 23.80% (398/1672) for ventriculoperitoneal shunt. The odds ratio for lumboperitoneal shunt versus ventriculoperitoneal shunt complication rates was 0.29 (95% CI 0.19 to 0.45, *p* < 0.0001), and the I^2^ was 72%. The shunt obstruction/malfunction rate was 3.99% (48/1204) for lumboperitoneal shunt and 8.31% (115/1384) for ventriculoperitoneal shunt (Odds ratio 0.54, 95% CI 0.37 to 0.79, *p* = 0.002, I^2^ = 0%). Based on the Modified Rankin Scale score, there were no differences in effectiveness between lumboperitoneal shunt and ventriculoperitoneal shunt. Nevertheless, lumboperitoneal shunt improved radiological outcomes.

**Conclusions:**

This analysis demonstrated that lumboperitoneal shunt is a safe and equally effective choice for treating communicating hydrocephalus. More studies are needed to confirm the safety of lumboperitoneal shunt.

## Graphical abstract

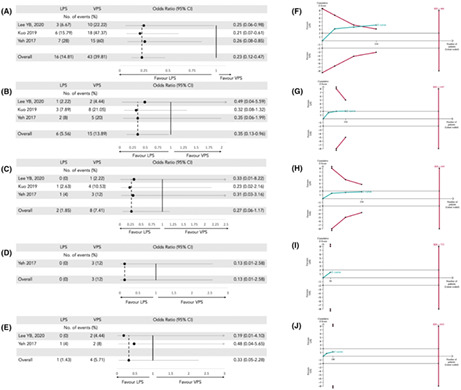

## 1 INTRODUCTION

Since the first hydrocephalus shunt in 1956, this effective surgical treatment has been developed with biocompatible material and valve system improvements.^1^ Ventriculoperitoneal shunt (VPS) implantation is the standard surgical treatment for cerebrospinal fluid (CSF) diversion, especially in North America and Europe.^2^ Although VPS provides effective CSF diversion and immediate symptomatic improvement, complications due to the intracranial placement, including brain hemorrhages, brain damage, infections, coma and, rarely, death, may occur.^3^ Shunt malfunction and complications causing high revision rate is also a risk.^4^ Although LPS has been available for 60 years, this shunt has not gained the same status. LPS provides an alternative for patients with communicating hydrocephalus.^5^ The use of LPS has increased in recent years due to the avoidance of brain damage and extracranial access. LPS is the most popular treatment for idiopathic normal pressure hydrocephalus (iNPH) in Japan.^6^, ^7^

The new LPS design, including a programmable valve setting, may provide better safety and lower adverse effects than VPS for patients with hydrocephalus.^8^ Only a few studies have compared the complications and efficacy of the LPS and VS. Therefore, we conducted a systematic review and meta‐analysis to compare the safety and adverse effects of LPS and VPS in patients with communicating hydrocephalus. We also compared clinical and radiological improvements after treatment with LPS and VPS.

## 2 METHODS

The meta‐analysis followed the reporting guidelines of the Preferred Reporting Items for Systematic Reviews and Meta‐Analyses (PRISMA)^9^ report. We included randomized controlled trials (RCTs), non‐RCTs and retrospective studies. Although we intended to enroll only RCTs, these trials were scarce. Therefore, we included nonrandomized concurrent trials and retrospective studies in the analysis.

### 2.1 Search strategy

We searched the following sources for eligible reports in any language: the Cochrane Library, PubMed, Embase, ClinicalTrials.gov, Cochrane Central Register of Controlled Trials, WanFang database and the China National Knowledge Infrastructure database. The search using free texts and medical subject headings included “hydrocephalus,” “communicating hydrocephalus,” “lumboperitoneal shunt,” “ventriculoperitoneal shunt,” “shunt,” “complications,” “adverse events,” and “efficacy.” Two review authors (YJ Ho, WC Chiang) independently searched the databases. We identified other potentially eligible trials, studies or ancillary publications by searching the reference lists of the retrieved trials, reviews and meta‐analyses. We also searched gray literature on Open Gray.

### 2.2 Inclusion/exclusion criteria

We included studies with a head‐to‐head comparison between VPS and LPS. We reviewed studies with clinical and imaging diagnoses of communicating hydrocephalus, all causes and subtypes of communicating hydrocephalus, and studies that analyzed the primary and secondary outcomes listed below.

#### 2.2.1 Primary outcomes

- Treatment failure: defined as morbidity associated with shunt placement (obstruction, over drainage, or infection)
- Adverse events: seizure and intracranial or intra‐abdominal hemorrhage (subdural hematomas, intraventricular hematomas, subarachnoid hematomas, or peritoneal end hematomas)

#### 2.2.2 Secondary outcomes

- Neurological disability improvement measured according to a validated score, such as the Modified Rankin Scale (mRS)
- Radiological outcome assessment with ventricular size reduction measured by cranial computed tomography (CT) scan or magnetic resonance imaging (MRI)

### 2.3 Selection of studies and data extraction and management

Two review authors (YJ Ho and WC Chiang) independently extracted data using data collection forms designed to capture information specific to this review. We performed the meta‐analysis in R (R Core Team (2021). R: A language and environment for statistical computing. R Foundation for Statistical Computing, Vienna, Austria.), RStudio (RStudio Team (2021). RStudio: Integrated Development Environment for R. RStudio, PBC, Boston, MA URL), using a random‐effects model and reporting 95% confidence intervals. Mantel and Haenszel^10^ pooling methods was applied in studies including only randomized controlled trials. When including nonrandomized studies, generic inverse variance method was used. The ratio measures of intervention effect were transformed into natural logarithms before analysis. We defined statistically significant differences as *p* < 0.05. The restricted maximum likelihood estimator^11^ was used to calculate the heterogeneity variance τ^2^ in continuous outcomes and the DerSimonian‐Laird estimator^12^ in binary outcomes. We used Knapp‐Hartung adjustments (Knapp & Hartung)^13^ to calculate the confidence interval around the pooled effect. Zero cells were dealt with using a continuity correction by Gart and Zweifel.^14^ We performed a sensitivity analysis using a Bayesian approach with the Markov Chain Monte Carlo method. We present the Doi plot with the Luis Furuya‐Kanamori index^15^ for each endpoint for publication bias.

### 2.4 Assessment of risk of bias in included studies

Two review authors independently assessed the risk of bias of the included studies. We used the Cochrane “RoB 2” assessment tool for randomized trials and ROBINS‐1 for nonrandomized studies.

### 2.5 Trial sequential analysis

A trial sequence analysis (TSA) was employed to quantify the statistical reliability of data through repetitive and cumulative testing. The TSA was conducted using TSA software (version 0.9.5.10 beta, Copenhagen Trial Unit, Center for Clinical Intervention Research, Rigshospitalet). Type I and Type II errors were 5 and 20%, respectively, in the model. We used O'Brien‐Fleming monitoring boundaries for hypothesis testing. The cumulative effect of TSA was considered true positive if the Z‐curve crossed the O'Brien‐Fleming monitoring boundaries and considered true negative if the Z‐curve entered the futility area. The underpowered total sample size did not achieve the required information size. The intervention incidence and the control arms were determined from all of the enrolled studies.

### 2.6 Grading of the certainty of evidence

We evaluated every result in the RCT subgroups using the Grading of Recommendations, Assessment, Development and Evaluation (GRADE)^16^ methodology. The overall certainty of evidence (CoE) was judged by five downgrading and three upgrading domains. The level of CoE was classified as high, moderate, low or very low.

## 3 RESULTS

### 3.1 Literature search

**FIGURE 1:**
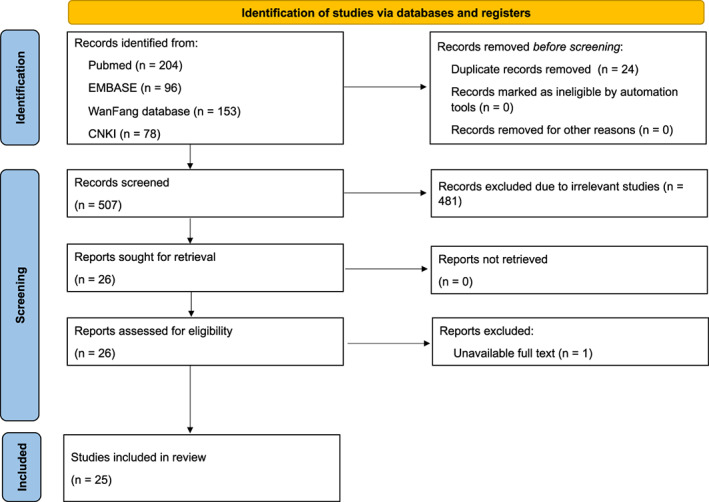
Flow diagram of preferred reporting items for systematic reviews and meta‐analysis (PRISMA) 2020.

The study selection process flow diagram is presented as a PRISMA flowchart (Figure 1). From the initial literature search, we retrieved 531 articles; 507 of these studies were duplicates or irrelevant. A total of 25 studies were identified. Manual searching of the reference lists of these studies did not yield new eligible studies. The characteristics of the included studies are presented in Table 1.

**TABLE 1:** Characteristics, description of patients, interventions, and endpoints of included studies

Study	Country	Age	Patient characteristics	Total number enrolled	Outcomes	Study design
Aoki et al., 1990^20^	Japan	1 mo–78 y/o	Hydrocephalus	LPS (*n* = 207) vs. VPS (*n* = 120)	Complications	Retrospective
Kang et al., 2000^47^	Korea	32–72 y/o	post‐SAH hydrocephalus	LPS (*n* = 22) vs. VPS (*n* = 34)	Stein and Langfitt grade	Retrospective
Huang et al., 2012^49^	China	19–68 y/o	Communicating hydrocephalus	LPS (*n* = 52) vs. VPS (*n* = 92)	Clinical improvement, complications	Retrospective
Singh et al., 2013^41^	India	Below 12 y/o	Postmeningitis communicating hydrocephalus	LPS (*n* = 37) vs. VPS (*n* = 53)	Complications	Retrospective
Yeh, 2014^34^	China	15–70 y/0	Communicating hydrocephalus	LPS (*n* = 58) vs. VPS (*n* = 70)	Complications, surgical successful rate at the first time	Retrospective
Ding et al., 2014^53^	China	15–70 y/o	Post‐traumatic hydrocephalus	LPS (*n* = 70) vs. VPS (*n* = 70)	Clinical status defined by James H. Salmon	Retrospective
Dong, 2015^51^	China	31–68 y/o	post‐SAH communicating hydrocephalus	LPS (*n* = 18) vs. VPS (*n* = 18)	Clinical symptoms improvement, radiological improvement, complications	Retrospective
Sosin et al., 2015^40^	U.S.	16–83 y/o	Communicating hydrocephalus	Laparoscopic‐assisted LPS (*n* = 9) vs. Laparoscopic‐assisted VPS (*n* = 44)	Intraoperative outcomes, length of stay, complications	Retrospective
Wang et al., 2015^39^	China	16–72 y/o	Post‐traumatic hydrocephalus	LPS (*n* = 52) vs. VPS (*n* = 65)	Clinical symptoms, GCS	Retrospective
Wu, 2015^37^	China	0.1–67 y/o	Communicating hydrocephalus	LPS (*n* = 15) vs. VPS (*n* = 14)	Radiological improvement, complications, NIHSS	Retrospective
Miyajima et al., 2016^29^	Japan	60–85 y/o	iNPH	LPS (*n* = 83) vs. VPS (*n* = 100)	mRS score 1 year after surgery, iNPHGS	Prospective
Kung et al., 2016^51^	China	11–78 y/o	Post‐traumatic hydrocephalus	LPS (*n* = 65) vs. VPS (*n* = 85)	Clinical symptoms improvement, radiological improvement, complications	Retrospective
Yan et al., 2016^36^	China	22–65 y/o	Communicating hydrocephalus	LPS (*n* = 47) vs. VPS (*n* = 143)	Complications	Retrospective
Yeh, 2017^33^	China	31–78 y/o	Communicating hydrocephalus	LPS (*n* = 25) vs. VPS (*n* = 25)	Complications, NIHSS	RCT
Lu et al., 2017^43^	China	Average 48.95 y/o	Post‐traumatic hydrocephalus	LPS (*n* = 40) vs. VPS (*n* = 40)	China Stroke Scale, ICI‐Q‐SF, radiological improvement	Retrospective
Lee et al., 2018^46^	China	Average 45.5 y/o	Communicating hydrocephalus	LPS (*n* = 20) vs. VPS (*n* = 20)	Operating time, postoperative to off the bedtime, length of stay, complications	RCT
Pang et al., 2018^42^	China	<1–>60 y/o, average 39.06 y/o	Communicating hydrocephalus	LPS (*n* = 18) vs. VPS (*n* = 46)	Operating time, use of postoperative antibiotics time, complications	Retrospective
Yang et al., 2019^35^	Taiwan	Not mentioned	Communicating hydrocephalus	LPS (*n* = 96) vs. VPS (*n* = 192)	Revision, complications	Retrospective
Wang et al., 2019^38^	China	42–61	Posthemorrhagic hydrocephalus	LPS (*n* = 56) vs. VPS (*n* = 102)	Clinical improvement, radiological improvement, GCS, EI, complications	Retrospective
Chen et al., 2019^54^	China	Above 60 y/o	iNPH (triad)	LPS (*n* = 28) vs. VPS (*n* = 68)	Triad symptoms improvements, complications	Retrospective
Jie, 2019^48^	China	18–64 y/o	Communicating hydrocephalus	LPS (*n* = 30) vs. VPS (*n* = 30)	Clinical improvement, radiological improvement, complications, surgical successful rate at the first time, NIHSS	Retrospective
Kuo et al., 2019^50^	China	31–64 y/o	Communicating hydrocephalus	LPS (*n* = 38) vs. VPS (*n* = 38)	Clinical improvement, radiological improvement, complications	RCT
Lee et al., 2020^44^	China	30–60 y/o	Communicating hydrocephalus	LPS (*n* = 45) vs. VPS (*n* = 45)	Clinical improvement, radiological improvement, complications	RCT
Lee et al., 2020^45^	China	8–74 y/o	Communicating hydrocephalus	LPS (*n* = 58) vs. VPS (*n* = 50)	Clinical improvement, radiological improvement, complications	Retrospective

### 3.2 Included studies

Three studies were RCTs, one was a prospective nonrandomized trial, 20 were retrospective studies and one was a nationwide epidemiological survey. Participants were diagnosed with communicating hydrocephalus by clinical symptoms and image studies (CT or MRI). The detailed surgical techniques were slightly different but included no differences that would alter the results among studies, except one study focusing on laparoscopy‐assisted VPS and LPS. The outcomes included clinical symptoms, intraoperative parameters, perioperative parameters, length of hospital stay, radiological improvement, complications, National Institutes of Health Stroke Scale/Score, mRS and the Stein and Langfitt hydrocephalus grade.

### 3.3 Description of studies

LPSs were used in 1189 patients. The studies were published between 1990 and 2020. Five studies were conducted in the United States, Japan and Korea, and the remaining 20 studies were in developing countries, including India and China. The shunting effectiveness was assessed using the mRS in 2 studies and radiological outcome improvement on follow‐up images (CT or MRI) in 11 studies. Total complications were reported in 21 studies, infection rates in 20 studies, seizure rates in 11 studies, shunt obstructions in 17 studies and hemorrhage rates in 13 studies.

### 3.4 Risk of bias in included studies

**FIGURE 2:**
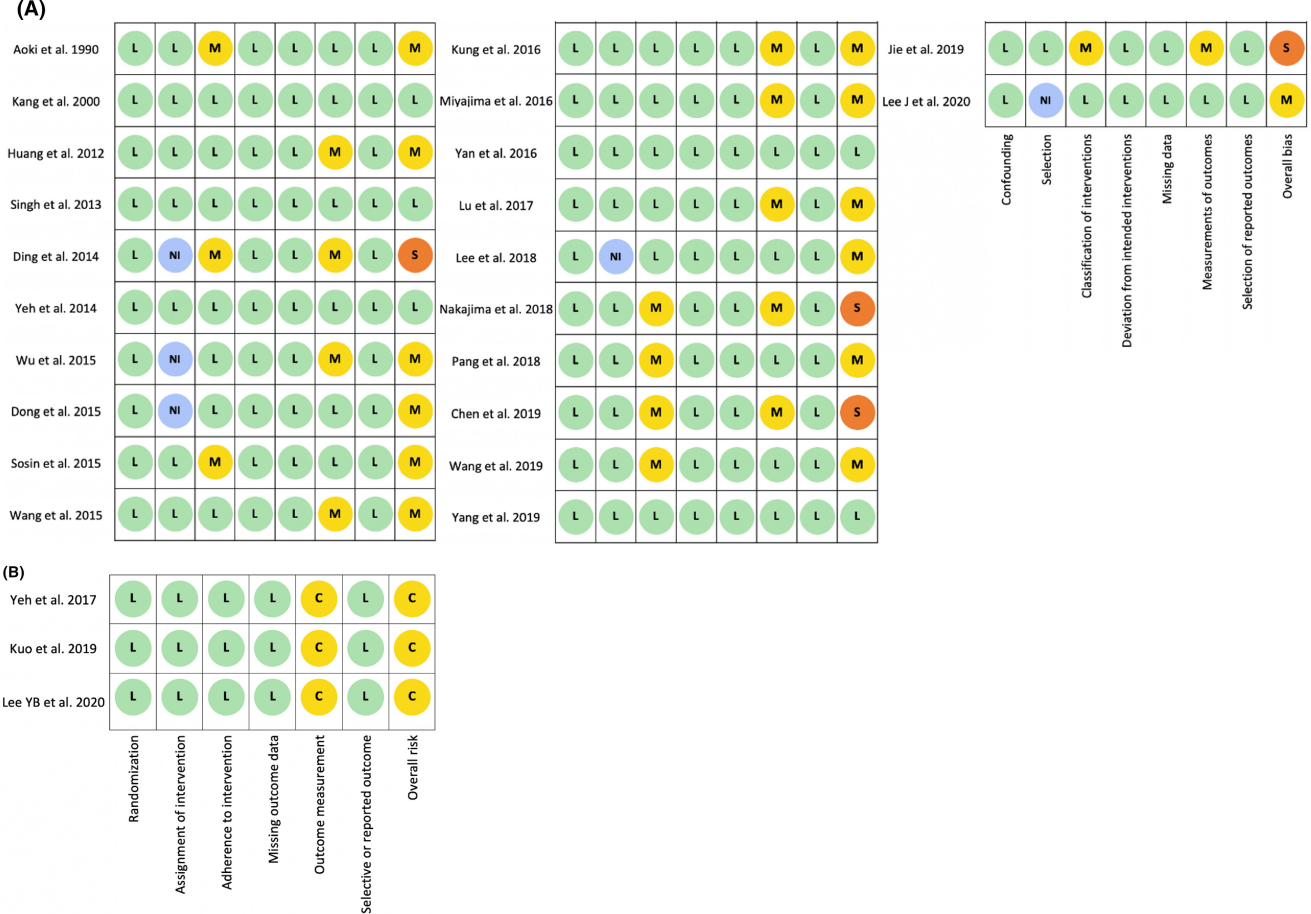
Risk‐of‐bias assessment on (A) nonrandomized studies; (B) randomized controlled trials.

All three RCTs had “some concerns.” In the domain of outcome measurement, three RCTs had “some concerns” due to the lack of blinded assessors. Only five nonrandomized studies were at a “low” overall risk of bias; 13 nonrandomized studies were judged as “moderate” risk of bias and four were at “serious” overall risk of bias (Figure 2).

### 3.5 Meta‐analysis of all studies

Data from 25 RCTs, non‐RCTs, prospective cohorts and retrospective studies, including 3654 patients, were analyzed. LPS was associated with a lower incidence of total complications compared with the incidence in VPS. There was low heterogeneity across the included studies. The total complication rate was 12.98% (188/1448) for LPS and 23.80% (398/1672) for VPS. The odds ratio was 0.29 (95% CI 0.19 to 0.45, *p* < 0.0001) and the I^2^ was 72% (Figures S1–S7).

#### 3.5.1 Primary outcomes: treatment failure

The shunt obstruction/malfunction rate was 3.99% (48/1204) for LPS and 8.31% (115/1384) for VPS. The odds ratio was 0.54 (95% CI 0.37 to 0.79, *p* = 0.002). The I^2^ was 0% (Figure S1B).

#### 3.5.2 Primary outcomes: adverse events

The infection rate for LPS was 1.53% (24/1568), which was significantly lower than the infection rate for VPS of 5.41% (97/1792). The odds ratio was 0.33 (95% CI 0.20 to 0.52, *p* < 0.0001) and I^2^ was 0% (Figure S1C). The seizure rate was 0.21% (2/961) for LPS and 2.57% (29/1129) for VPS. The odds ratio was 0.49 (95% CI 0.21 to 1.13) and the I^2^ was 0% (Figure S1D). The hemorrhage rate was 2.4% for LPS (33/1375) and 5.03% (77/1532) for VPS, with an odds ratio of 0.57 (95% CI 0.36 to 0.89, *p* = 0.01) and an I^2^ of 0% (Figure S1E).

#### 3.5.3 Secondary outcomes: neurological disability improvement and radiological outcome assessment

No differences in effectiveness between LPS and VPS were detected, based on the mRS score (Figure S2A). However, radiological outcomes improvement rate was better after LPS than VPS (Figure S2B).

### 3.6 RCT subgroup analysis

**FIGURE 3:**
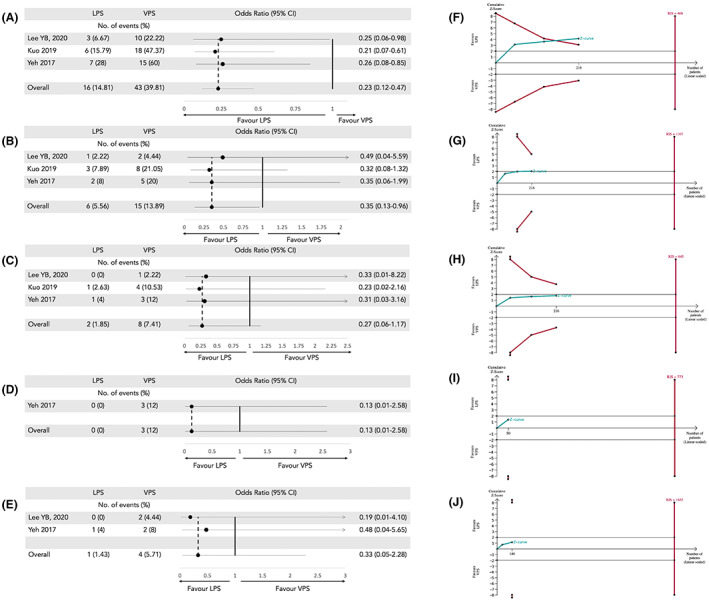
Forest plot (A–E) and trial sequential analysis (F–J) of total complication rate (A, F), shunt obstruction/malfunction rate (B, G), infection rate (C, H), seizure rate (D, I) and hemorrhage rate (E, J) between lumboperitoneal shunt (LPS) and ventriculoperitoneal shunt (VPS), respectively. CI, confidence interval; RIS, required information size.

We conducted RCT subgroup analyses for total complications rate, infection rate, shunt obstruction rate, seizure rate, hemorrhage rate and radiological improvement. The total complication rate was lower for LPS (14.81%, 16/108) than VPS (39.81%, 43/108). The odds ratio was 0.23 (95% CI 0.12 to 0.47, *p* < 0.0001) and the I^2^ was % (Figure 3A).

#### 3.6.1 Primary outcomes: treatment failure

The shunt obstruction/malfunction rate was 5.56% (6/108) for LPS and 13.89% (15/108) for VPS. The odds ratio was 0.35 (95% CI 0.13 to 0.96, *p* = 0.04) and the I^2^ was 0% (Figure 3B).

#### 3.6.2 Primary outcomes: adverse events

The infection rate for LPS was 1.85% (2/108), lower than the infection rate for VPS (7.41%, 8/108); however, the difference was not statistically significant. The odds ratio was 0.27 (95% CI 0.06 to 1.17, *p* = 0.08) and the I^2^ was 0% (Figure 3C). The seizure rate was 0% (0/25) for LPS and 12% (3/25) for VPS. However, seizure rates were only reported in one study (Figure 3D). The hemorrhage rate was 1.43% for LPS (1/70) and 5.71% (4/70) for VPS. The odds ratio was 0.33 (95% CI 0.05 to 2.28, *p* = 0.26) and the I^2^ = 0%. The difference was not statistically significant (Figure 3E).

#### 3.6.3 Secondary outcomes: neurological disability improvement and radiological outcome assessment

**FIGURE 4:**
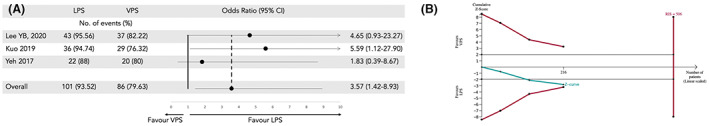
Forest plot (A) and trial sequential analysis (B) of radiological improvement rate. CI, confidence interval; RIS, required information size.

No RCT investigated neurological disability improvements. Radiological improvement rates among RCTs was 93.52% (101/108) for LPS and 79.63% (86/108) for VPS, with an odds ratio of 3.57 (95% CI 1.42 to 8.93, *p* = 0.007). The I^2^ was 0% (Figure 4A). The cumulative Z‐curve crossed conventional test boundary but not yet O'Brien‐Fleming monitoring boundary. (Figure 4B).

### 3.7 Publication bias

A review of the Doi plots with the Luis Furuya‐Kanamori index for each endpoint could not exclude the potential for publication bias for, total complication rates (Figure S3), shunt obstruction/malfunction rates (Figure S4), hemorrhage rates (Figure S5) and radiological improvement rate (Figure S6). The results did not change between fixed or random‐effects models. Doi plots also validated publication bias in RCT subgroup. Publication bias was strongly suspected in hemorrhages (Figure S7).

### 3.8 Trial sequential analysis

TSAs were conducted for all RCT subgroups' endpoints. The cumulative Z‐curve crossed O'Brien‐Fleming monitoring boundaries favor LPS for lower total complications. Diversity = 0%. (Figure 3F) However, in the shunt obstruction/malfunction analysis, the Z‐curve passed the conventional boundary favoring LPS (*p* = 0.04). Diversity = 0% (Figure 3G). After correction, the TSA did not pass the trial sequential monitoring boundary and the total number of patients did not reach the required information size. From this perspective, the analysis was statistically inconclusive. In the infection, seizure and hemorrhage analysis, the cumulative Z‐curve did not pass conventional test boundary either O'Brien‐Fleming monitoring boundaries. (Figure 3H‐J).

## 4 DISCUSSION

Our meta‐analysis demonstrated that adverse effects occur less frequently after LPS implantation than after VPS implantation for patients with communicating hydrocephalus without compromising the effectiveness of treatment. We compared the safety, neurologic disability and radiological improvement between the two shunts. Contrary to our initial hypothesis, the two shunts had similar safety and effectiveness. LPS appeared to be better when comparing total complications, including infections, seizures, shunt obstructions and hemorrhage. The use of LPS also resulted in better radiological outcomes. The results from the RCT subgroup analysis, which TSA verified, also favored LPS, which yielded lower total complication rates. Despite still not reaching the required information size, the cumulative z‐curve will need to pass through the futility area to reach the area favoring VPS, leaving little chance to overthrow the hypothesis that LPS is superior than VPS as far as complication rates are concerned.

To the best of our knowledge, this meta‐analysis is the first report to demonstrate decreased complications in patients treated with LPS. The total complication rate for LPS was 12.98%, and the total complication rate for VPS was 23.80%. Previous studies also revealed similar complication rates of VPS ranging from 13% to 38%, which mostly occurred in the first year after surgery.^17^, ^18^ The infection rate for LPS (1.53%) was lower than the infection rate for VPS (5.41%). Infection rates varied between studies, from 1% to 9%.^19^, ^20^ Obstruction/malfunction rates were also lower for LPS (3.99%) than VPS rates (8.31%). Aoki et al.^20^ demonstrated that infection and malfunction rates after LPS implantation were significantly lower than those of VPS. Yadav et al.^19^ demonstrated a lower incidence of shunt obstruction for LPS than shunt obstruction for VPS.

Intracranial access is unnecessary during LPS implantation, which may lower the risk of intraparenchymal hemorrhage. The hemorrhage rate for LPS was 2.40%, which was significantly lower than the VPS hemorrhage rate of 5.03%. However, some studies included in our analysis did not specify the type of hemorrhage. Different types of hemorrhages are thought to be caused by different etiologies. For instance, chronic subdural hemorrhage may be caused by overshunting and intraparenchymal hemorrhage or intraventricular hemorrhage may be caused by ventricular puncturing. Seizure rates were also higher in VPS, which may be due to puncturing through the cerebral cortex and other complications, such as lung or diaphragm injury, during subcutaneous tunneling.^21^ Although rare, over drainage causing slit ventricle syndrome, intracranial hypotension syndrome, chronic subdural effusion, or subdural hemorrhage may also be the disadvantages of VPS.^22^ On the other hand, the lumbar exit and peritoneal entry for LPS are generally at the same level when the patients are upright to minimize the effect of gravity, and the siphoning effect is negligible.^23^, ^24^

In our analysis, 75.81% of patients had different degrees of radiological improvements after shunting. However, improvements were defined differently in different studies. The results are similar to previous studies, which showed that more than 75% of patients showed improvement after shunting.^25^, ^26^, ^27^ However, our study results indicated a significantly better outcome for LPS. The improved outcome may be due to the increased compatibility with CSF dynamics. More studies are needed to verify this result due to vague or inconsistent radiological improvements among different studies.

Several studies reported the safety and noninferior effectiveness of LPS compared to VPS in iNPH.^25^, ^28^, ^29^, ^30^, ^31^, ^32^ Kazui et al.^25^ reported that LPS is a safe and beneficial treatment option for iNPH. Miyajima et al.^8^ showed that the efficacy and safety of LPS with programmable valves are comparable to those of VPS for the treatment of iNPH. Bloch et al.^30^ demonstrated that gait improved in 33/33 (100%) patients, incontinence improved in 13/28 (46%) patients and memory improved in 11/20 (55%) patients after LPS placement. The analysis by Giordan et al.^32^ suggested that outcomes for iNPH did not change significantly between VPS and LPS.

### 4.1 Limitations

We aimed to include many patients for the meta‐analysis to compare outcomes between patients with hydrocephalus undergoing VPS and LPS. However, there were several limitations to this study. First, most studies included in the analysis were retrospective, nonrandomized trials. Although three studies were RCT trials, the small number of participants and the low number of events constrained the study. However, the results of the TSA confirmed the decreased total complication rate in patients receiving LPS as a treatment for hydrocephalus. Second, idiopathic and secondary communicating hydrocephalus were not discussed separately in our analysis, resulting in a different outcome. Third, we did not perform a meticulous analysis of different valve types, which may give us a closer look at the complications. Some of the valve systems were programmable and more flexible. Fourth, most of the studies included were performed in Asia, except one in the USA. All three randomized controlled trials were also conducted in Asia. The subjects included in these studies were mostly Asians, and the lack of variety of ethnicity could bias the results. We included these studies hosted by different hospitals. They all might have different protocols for performing VPS and LPS surgeries. It could be another bias but could not be avoided easily, especially when conducting a meta‐analysis that tends to include more data based on inclusion criteria and primarily discusses outcomes between two different surgical procedures. Fifth, one of the crucial complications, overdrainage, should have been discussed. Unfortunately, only five studies included in this meta‐analysis mentioned overdrainage but all without a clear definition. Moreover, some of them combined overdrainage with underdrainage into a category. We chose not to pool these outcomes together due to their ambiguity. However, it was indeed a vital topic that should be discussed. More studies are expected to give us more insight into it. Last, our results are rated low and very low CoE when evaluated with GRADE methodology (Table 2). Again, more high‐quality and significant studies are needed.

**TABLE 2:** GRADE assessment.

Lumboperitoneal shunts compared to ventriculoperitoneal shunts for communicating hydrocephalus							
Certainty assessment							Summary of findings
Participants (studies) follow‐up	Risk of bias	Inconsistency	Indirectness	Imprecision	Publication bias	Overall certainty of evidence	Anticipated absolute effects: mean difference/risk difference
Improvements, mRS							
1236 (3 non‐RCTs)	Very serious	Not serious	Not serious	Not serious	Publication bias unlikely	Low ⊕⊕⊝⊝	No difference
Improvements, radiological outcomes							
216 (3 RCTs)	Serious	Not serious	Not serious	Serious	Publication bias unlikely	Low ⊕⊕⊝⊝	139 more per 1000
Total complications							
216 (3 RCTs)	Serious	Not serious	Not serious	Serious	Publication bias unlikely	Low ⊕⊕⊝⊝	250 fewer per 1000
Infection							
216 (3 RCTs)	Serious	Not serious	Not serious	Serious	Publication bias unlikely	Low ⊕⊕⊝⊝	No difference
Seizure							
2090 (1 RCT, 12 non‐RCTs)	Very serious	Not serious	Not serious	Not serious	Publication bias unlikely	Low ⊕⊕⊝⊝	No difference
Shunt obstruction/malfunction							
216 (3 RCTs)	Serious	Not serious	Not serious	Serious	Publication bias unlikely	Low ⊕⊕⊝⊝	83 fewer per 1000
Hemorrhage							
140 (2 RCTs)	Serious	Not serious	Not serious	Serious	Publication bias strongly suspected	Very low ⊕⊝⊝⊝	No difference

## 5 CONCLUSION

Our meta‐analysis indicates that LPS is a safe and equally effective treatment for hydrocephalus compared with VPS. LPS had a lower complication rate, including lower infection, seizure, shunt obstruction/malfunction and hemorrhage rate, compared to VPS. Suppose more high‐quality studies in the future confirm these beneficial results. In that case, LPS could be a good alternative to VPS or even a first‐line treatment option for patients with communicating hydrocephalus who are not a good candidate for VPS.

## AUTHOR CONTRIBUTIONS

Yi‐Jen Ho involved in conceptualization, data curation, formal analysis, methodology, software, visualization, writing the original draft, review, and editing. Wen‐Chun Chiang involved in data curation, formal analysis, methodology, and software. Hsin‐Yi Huang and Shinn‐Zong Lin involved in supervision. Sheng‐Tzung Tsai involved in supervision, review, and editing.

## CONFLICT OF INTEREST

The authors declare that they have no conflict of interests and have no funding to support this study.

## Supporting information


Figures S1–S7

## Data Availability

The data that supports the findings of this study are available in the supplementary material of this article.
