# Supplementary material for: Effectiveness and safety of ventriculoperitoneal shunt versus lumboperitoneal shunt for communicating hydrocephalus: A systematic review and meta‐analysis with trial sequential analysis
Source: CNS Neurosci Ther. 2023 Jan 17;29(3):804–15. doi: 10.1111/cns.14086 (PMC9928545; doi:10.1111/cns.14086)

**Figures included in online-only material**

Supplementary Figure 1A

Supplementary Figure 1B

Supplementary Figure 1C

Supplementary Figure 1D

Supplementary Figure 1E

Supplementary Figure 2A

Supplementary Figure 2B

Supplementary Figure 3

Supplementary Figure 4

Supplementary Figure 5

Supplementary Figure 6

Supplementary Figure 7

**Supplementary Figure 1A**


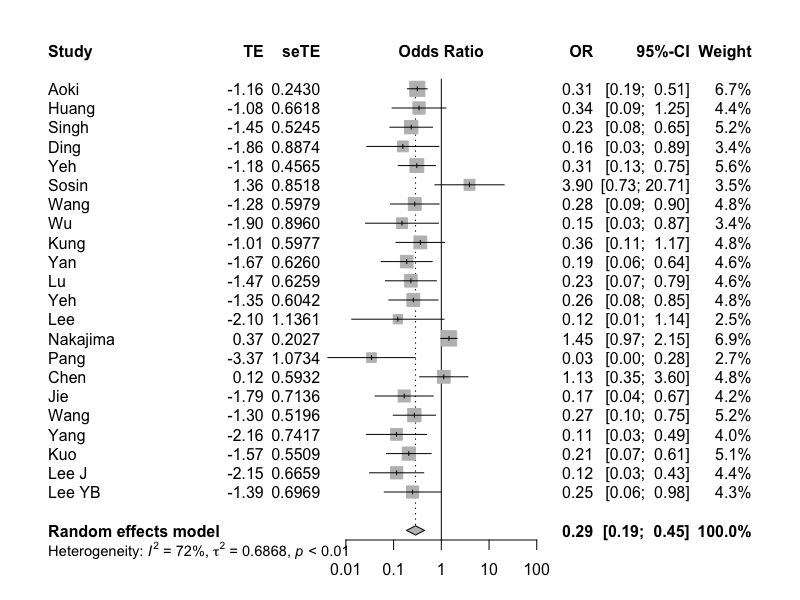


**Supplementary Figure 1B**

**
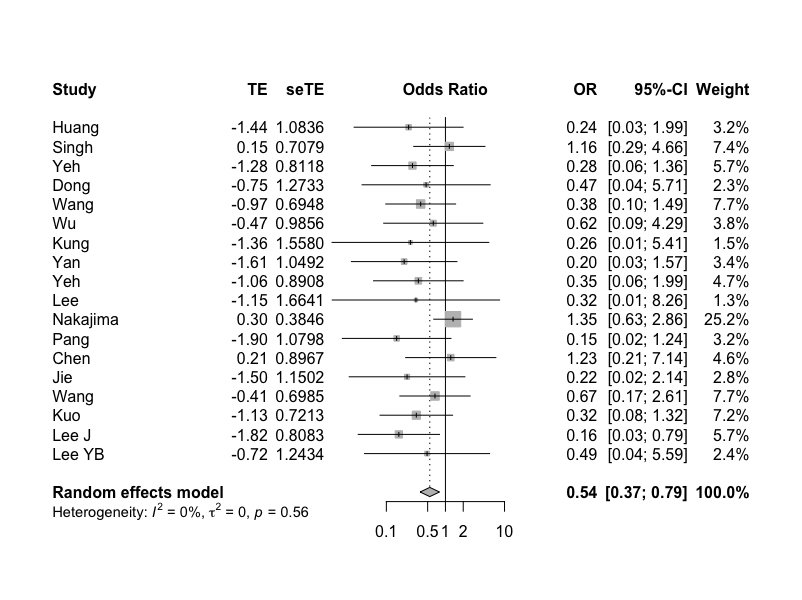
**

**Supplementary Figure 1C**

**
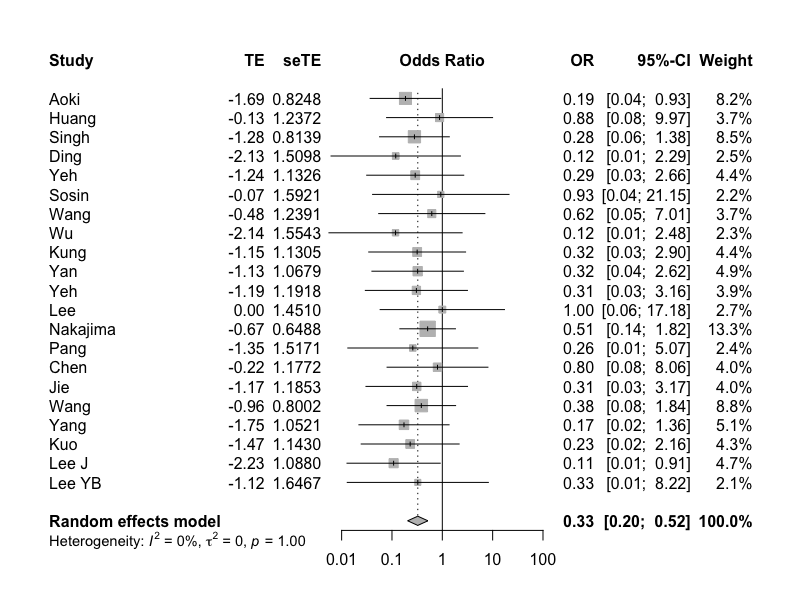
**

**Supplementary Figure 1D**

**
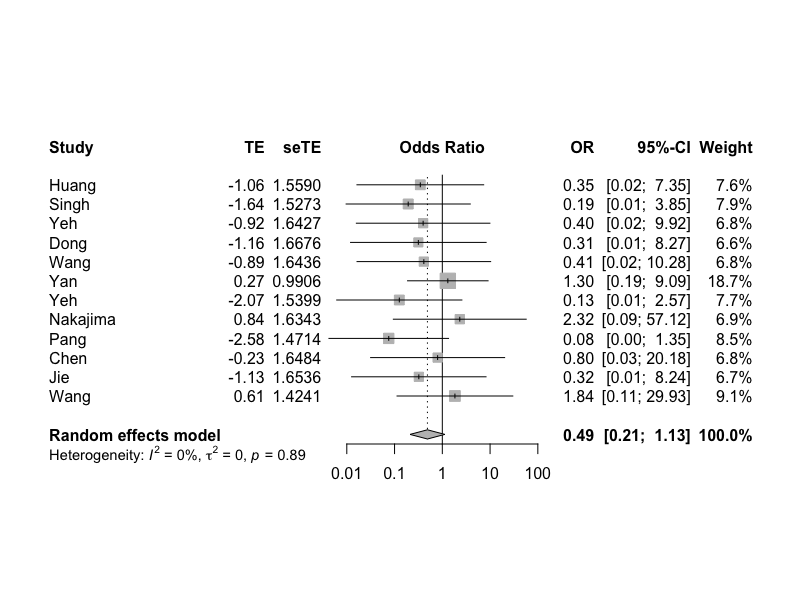
**

**Supplementary Figure 1E**

**
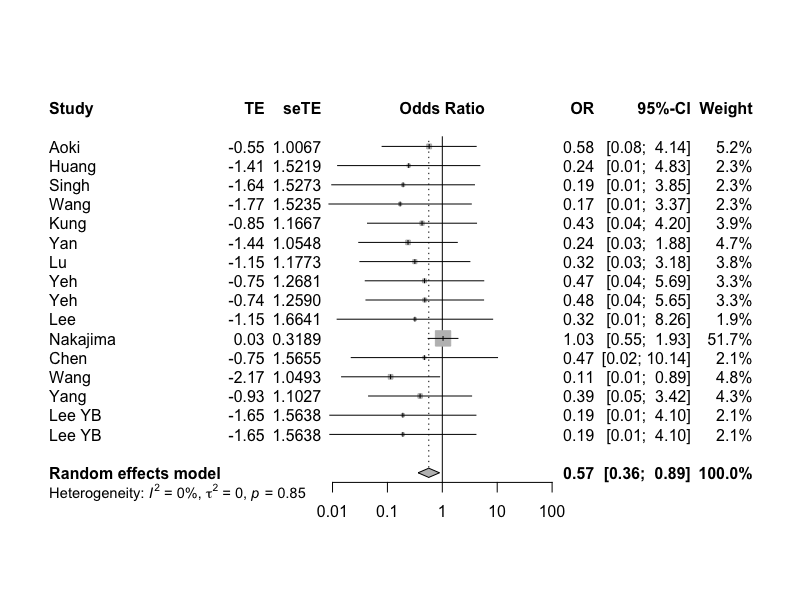
**

**Supplementary Figure 2A**


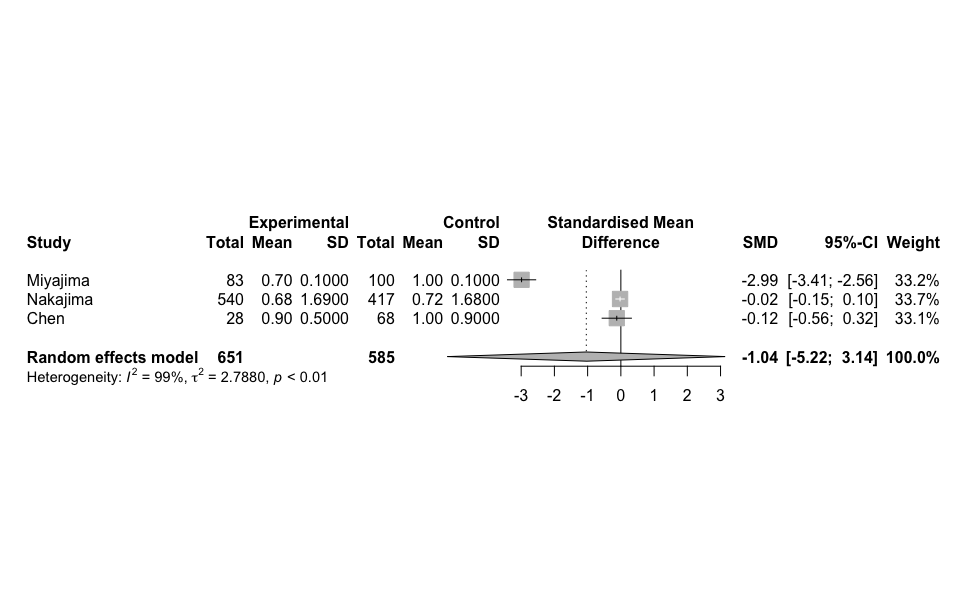


**Supplementary Figure 2B**

**
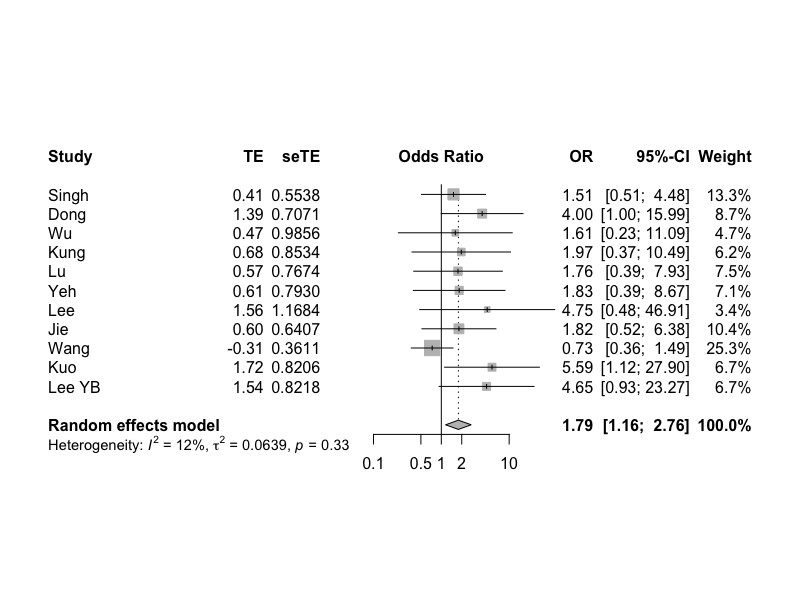
**

**Supplementary Figure 3**

**
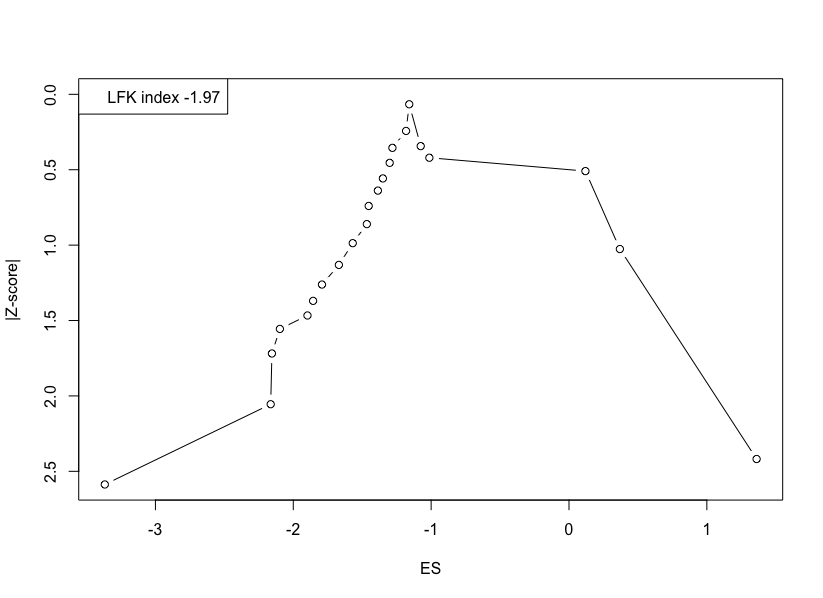
**

**Supplementary Figure 4**


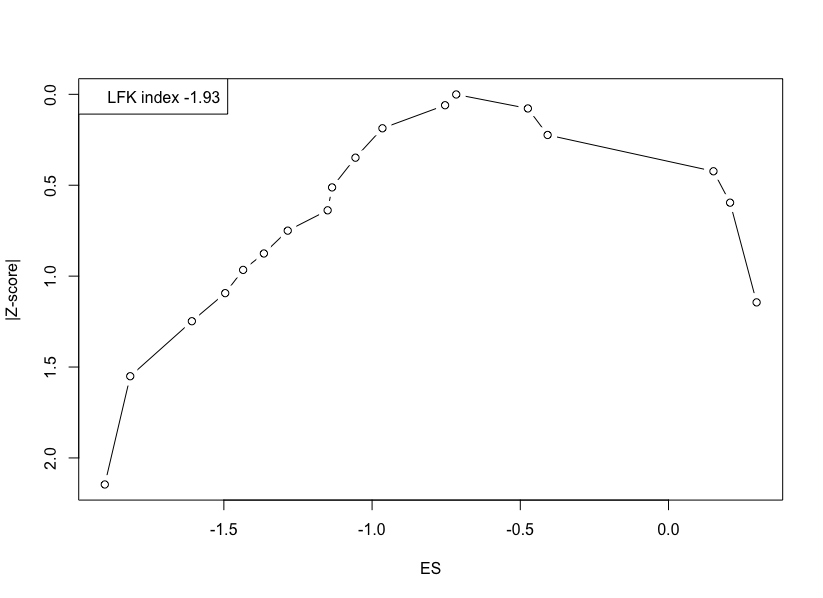


**Supplementary Figure 5**


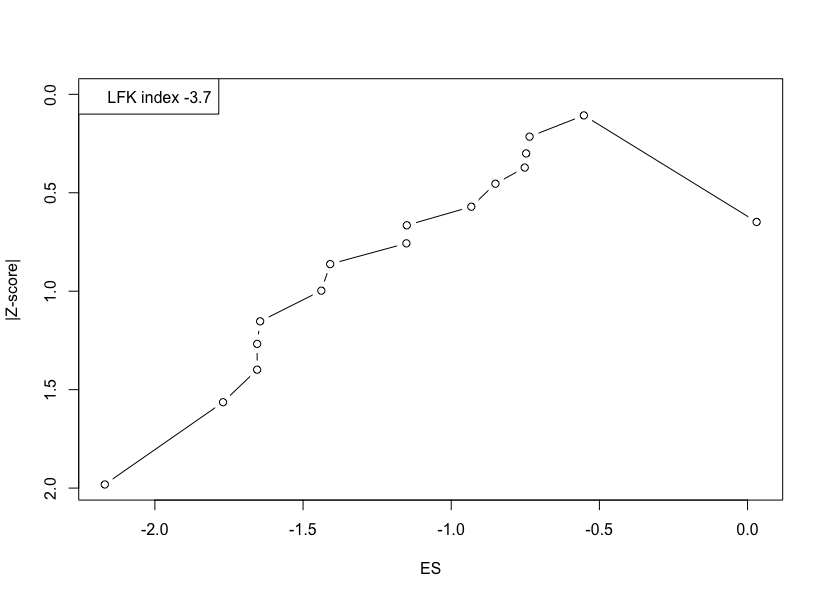


**Supplementary Figure 6**


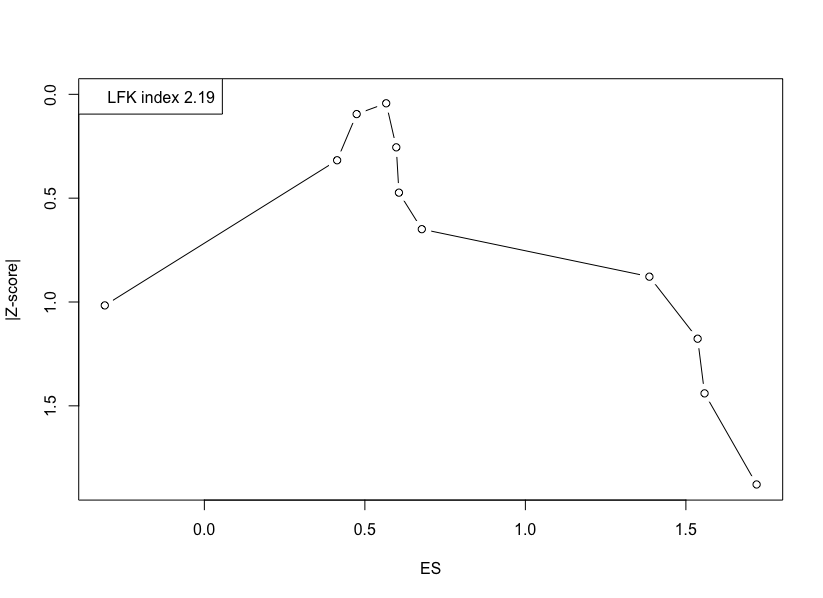


**Supplementary Figure 7**


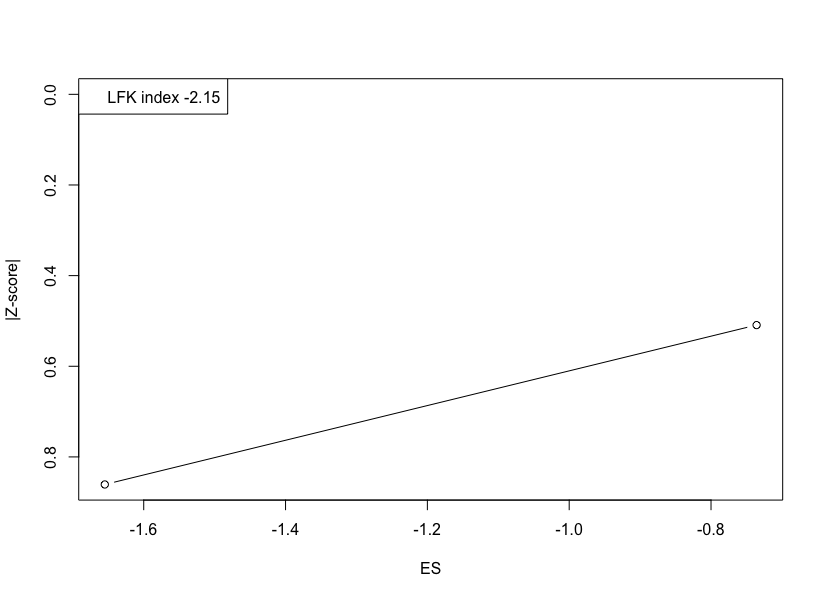

Supplement: Supplementary file 1 — Figures S1–S7 [file CNS-29-804-s001.docx]
